# Identification and validation of neurotrophic factor-related genes signature in HNSCC to predict survival and immune landscapes

**DOI:** 10.3389/fgene.2022.1010044

**Published:** 2022-11-04

**Authors:** Gaoge Peng, Hao Chi, Xinrui Gao, Jinhao Zhang, Guobin Song, Xixi Xie, Ke Su, Binyu Song, Jinyan Yang, Tao Gu, Yunyue Li, Ke Xu, Han Li, Yunfei Liu, Gang Tian

**Affiliations:** ^1^ Clinical Medical College, Southwest Medical University, Luzhou, China; ^2^ School of Stomatology, Southwest Medical University, Luzhou, China; ^3^ Department of Plastic Surgery, Xijing Hospital, Fourth Military Medical University, Xi’an, China; ^4^ Queen Mary College, Medical School of Nanchang University, Nanchang, China; ^5^ Department of General, Visceral, and Transplant Surgery, Ludwig-Maximilians-University Munich, Munich, Germany; ^6^ Department of Laboratory Medicine, The Affiliated Hospital of Southwest Medical University, Luzhou, China

**Keywords:** head and neck squamous cell carcinoma, neurotrophic factor, prognostic signature, nomogram, tumor microenvironment, immunotherapy

## Abstract

**Background:** Head and neck squamous cell carcinoma (HNSCC) is the seventh most common type of cancer worldwide. Its highly aggressive and heterogeneous nature and complex tumor microenvironment result in variable prognosis and immunotherapeutic outcomes for patients with HNSCC. Neurotrophic factor-related genes (NFRGs) play an essential role in the development of malignancies but have rarely been studied in HNSCC. The aim of this study was to develop a reliable prognostic model based on NFRGs for assessing the prognosis and immunotherapy of HNSCC patients and to provide guidance for clinical diagnosis and treatment.

**Methods:** Based on the TCGA-HNSC cohort in the Cancer Genome Atlas (TCGA) database, expression profiles of NFRGs were obtained from 502 HNSCC samples and 44 normal samples, and the expression and prognosis of 2601 NFRGs were analyzed. TGCA-HNSC samples were randomly divided into training and test sets (7:3). GEO database of 97 tumor samples was used as the external validation set. One-way Cox regression analysis and Lasso Cox regression analysis were used to screen for differentially expressed genes significantly associated with prognosis. Based on 18 NFRGs, lasso and multivariate Cox proportional risk regression were used to construct a prognostic risk scoring system. ssGSEA was applied to analyze the immune status of patients in high- and low-risk groups.

**Results:** The 18 NFRGs were considered to be closely associated with HNSCC prognosis and were good predictors of HNSCC. The multifactorial analysis found that the NFRGs signature was an independent prognostic factor for HNSCC, and patients in the low-risk group had higher overall survival (OS) than those in the high-risk group. The nomogram prediction map constructed from clinical characteristics and risk scores had good prognostic power. Patients in the low-risk group had higher levels of immune infiltration and expression of immune checkpoints and were more likely to benefit from immunotherapy.

**Conclusion:** The NFRGs risk score model can well predict the prognosis of HNSCC patients. A nomogram based on this model can help clinicians classify HNSCC patients prognostically and identify specific subgroups of patients who may have better outcomes with immunotherapy and chemotherapy, and carry out personalized treatment for HNSCC patients.

## 1 Introduction

Head and neck cancer is the seventh most common type of cancer in the world, with a high incidence in Southeast Asia, Brazil, and Central Europe (Kaidar-Person et al., 2018). An estimated 700,000 new cases in 2018 augur well for a serious prognosis, of which 350,000 are expected to be fatal (Bray et al., 2018). At present, the treatment of HNSCC has been based on various treatment methods, such as chemotherapy, radiotherapy, and photodynamic therapy, and the survival rate of HNSCC patients within 5 years after early disease treatment is 70–90% (Lim et al., 2017). However, due to its highly invasive and heterogeneous nature, the prognosis of patients with HNSCC remains poor (Liang et al., 2021). At the same time, most cases of HNSCC are diagnosed at an advanced stage with poor medical treatment and require surgery to dismember the organs needed to speak and swallow (Hashim et al., 2019). For individuals in countries with limited access to tertiary care centers, survival rates are 30%–40% (Sinha et al., 2003; Attar et al., 2010; Pruegsanusak et al., 2012; Nandakumar and Nandakumar, 2016). Although the recurrence rate is unacceptably high after the patient recovers. In fact, nearly half of oral cancer patients will have a recurrence (Kademani et al., 2005; Koo et al., 2006; Haddad and Shin, 2008), and the 5-year survival rate in this condition is 35%–45%, which is frustrating (Kademani et al., 2005; Bell et al., 2007). To quell these adverse consequences, and to recognize that HNSCC is one of the most inflammatory tumor microenvironments (TME) of all solid tumors, treatment of head and neck cancer has begun to shift to immunotherapy (Horton et al., 2019). Now immunotherapy has become a model for cancer treatment and has received widespread attention as a precision medicine program for the treatment of solid malignancies (Xie et al., 2017). Since risk stratification based solely on tumor size, lymph node and distant metastases (TNM staging), and histological grade are not sufficient to predict prognosis in patients with HNSCC, such as squamous cell carcinoma of the tongue *versus* squamous cell carcinoma of the oral cavity, therefore there is an urgent need for more accurate models that predict prognosis (Kim et al., 2017; Gao et al., 2022). Nerve growth (Tumor neurogenesis) in the tumor microenvironment has recently been shown to be critical for cancer progression. Neurotrophic factors such as nerve growth factor (NGF), and brain-derived neurotrophic factor (BDNF), are considered drivers of neurogenesis during development and regeneration, playing a key role in the crosstalk between tumor cells and nerves (Gao et al., 2018). Studies have shown that nerves release neurotransmitters to promote tumor growth, and tumors secrete neurotrophic factors from each other, stimulate nerve growth and tumor cells to stimulate proliferation, survival, migration, and/or invasion, and favor tumor angiogenesis, while neurotrophic growth factors secreted by cancer cells can also drive the growth of nerves in solid tumors (Jobling et al., 2015; Chopin et al., 2016; Griffin et al., 2018). The effect of growing nerves on tumors has also been studied in other cancers, such as tumor cells and nerve endings such as laryngeal cancer and colorectal cancer by secreting and absorbing neurotrophic factors; Causing peripheral invasion (PNI) and promoting tumor progression (Hou et al., 2021; Zhang et al., 2022a). Tumor denervation of prostate, stomach, and pancreatic cancers reduces tumor growth and invasion; The presence of nerves is associated with metastasis and increased tumor grading (Rowe et al., 2020). Some studies have shown that BDNF protects neuroblastoma cells from chemotherapeutic agent-induced cytotoxicity. In the Triple-Negative Breast Cancer (TNBC) brain metastasis model, BDNF was shown to autocrine regulate the expression of the BDNF-tumor cell trophic carnosine kinase receptor B (TrkB) gene, thereby increasing the migration activity of cells (Zimmer, 2021). Nerve growth factor (NGF) from cancer cells causes increased nerve density in the tumor microenvironment (Rowe et al., 2020), while nerve cells expressing nerve growth factor (NGF) receptors of NTRK1 (TRKA) and NGFR (p75NTR) were found, and it was thought that there was a correlation between a large amount of NGF produced by cancer cells and the presence of nerves (*p* = 0.02) (Griffin et al., 2020). NGF has a promoting effect on various cancers, and anti-NGF has been shown to reduce tumor proliferation (Ye et al., 2011). In addition, we also found that NGF has the potential to selectively affect the proliferation of breast cancer cells rather than normal breast epithelial cells, so NGF may be the best treatment target for specific cancer types; The effect of NGF on cancer cells varies depending on the expression status of TrkA and/or p75NTR and varies with the use of chemotherapy drugs, and may have a greater impact on immune or drug therapeutic effects (Noh et al., 2017). This neurotrophic effect of NGF in cancer may be associated with a large number of human malignancies as well as other neurotrophins and may have an effect on cancer pain (Griffin et al., 2018).

In recent years, with the development of molecular biology techniques and bioinformatics, new biomarkers have the potential to become effective and specific prognostic factors for different types of cancer, including HNSCC. As far as we know, although there are a large number of studies exploring the mechanism and role of neurotrophic factors in various cancers, research on determining the prognosis of HNSCC as a target for immunotherapy through neurotrophic factor-related genes is still a blank. In view of the fact that its value and mechanism in the diagnosis and prognosis of HNSCC have not yet been clarified, this study used the TCGA-HNSC dataset to comprehensively analyze the relationship between the expression differences of NFRGs and the prognosis of HNSCC and screened out 18 reliable NFRGs. On this basis, we further constructed a prognostic model based on NFRGs, made a risk-scoring formula, and analyzed the correlation between the prognosis model and the immune microenvironment, gene mutation burden, and immunosuppressive point therapy, as well as the sensitivity of chemotherapy drugs. Through the comprehensive analysis of genomic data and clinically relevant data, we aim to demonstrate the value of NFRGs in predicting the prognosis of patients with HNSCC and improving the diagnosis of patients with HNSCC, and exploring more effective personalized treatment options.

## 2 Materials and methods

### 2.1 Data sources

We downloaded the TCGA-HNSC cohort from the TCGA database (https://portal.gdc.cancer.gov/), which includes 502 HNSCC samples and 44 normal samples. Of these, 501 HNSCC samples with complete clinical information were included in the follow-up analysis. The sample size of HNSCC patients at the M stage varied greatly. This stage was consequently excluded from the analysis. Based on relevant clinical information, the HNSCC cohort was randomly divided into training risk groups and test risk groups using the cart R software package. The ratio is 7:3. The model is externally validated using the GSE41613 dataset collected in GEO (Gene Expression Omnibus) as a validation set (N = 97).

### 2.2 Model construction

The model was constructed using univariate Cox regression analysis to screen for prognostically associated neurotrophic factor-related genes in the HNSCC cohort. Subsequently, neurotrophic factor-related genes (*p* < 0.05) significantly associated with prognosis in patients with HNSCC were incorporated into the Least Absolute Shrinkage and Selection Operator (LASSO) COX regression models, and the key genes and their regression coefficients were determined using the R package “glmnet” (Friedman et al., 2010). The risk fraction is generated using the following formula: risk fraction = ExpressionmRNA1 × CoefmRNA1 + ExpressionmRNA2 × CoefmRNA2 +. . ExpressionmRNAn × CoefmRNAn。

### 2.3 Model formulas

The risk score of all patients is calculated according to the output model equation, and then the optimal cut-off value is calculated using the R packet “survminer” all HNSCC patients are divided into high-risk and low-risk groups, and the survival curves of high-risk and low-risk groups are plotted. PCA analysis using R software and “pec” R packages are used to calculate the c-index. Time-dependent ROC curve analysis was performed using the “survivalROC” R package to assess the predictive power of genetic traits.

### 2.4 Independent prognostic analysis and nomogram predictive model construction

Univariate Cox regression and multivariate Cox regression analysis were used to assess whether the risk score was an independent prognostic factor. Using the “rms” R packet, a line plot was constructed using risk score, age, tumor stage, and model gene expression to predict the overall survival at 1, 3, and 5 years in HNSCC patients in the TCGA dataset.

### 2.5 Immunoassay of risk signatures

Currently recognized methods, including XCELL (Aran et al., 2017; Aran, 2020), TIMER (Chen et al., 2018; Li et al., 2020), QUANTISEQ (Finotello et al., 2019; Plattner et al., 2020), MCPCOUNT (Dienstmann et al., 2019), EPIC (Racle et al., 2017), CIBERSORT (Chen et al., 2018; Zhang et al., 2022b) and CIBERSORT-ABS (Tamminga et al., 2020) is used to measure immune infiltration scores. Spearman correlation analysis was used to explore the correlation between risk fraction and immune cells. To distinguish the immune infiltrative status of patients in the high-risk and low-risk groups, we used a single-sample GSEA (ssGSEA) method to calculate the immune cell characteristics of patients with HNSCC. At the same time, we collected 19 inhibitory immune checkpoints with therapeutic potential from Auslander’s study to compare their differences between high- and low-risk groups (Auslander et al., 2018). We obtained the gene set associated with cancer-immune circulation from the website developed by Xu et al (http://biocc.hrbmu.edu.cn/TIP/) Xu et al (2018). and the gene set that was positively correlated with the clinical response to the anti-PD-L1 drug (atezolizumab) from the research features of Mariathasan (Mariathasan et al., 2018). Using the GSVA algorithm (Hänzelmann et al., 2013)to calculate the enrichment scores of genetic signatures positively correlated with the cancer immune cycle and immunotherapy between the high-risk and low-risk groups, the *p*-value <0.05 was considered to have a significant difference’. The ggcor'R software package is used to analyze the correlation between risk scores and the two genetic traits described above.

### 2.6 Somatic mutation analysis

We downloaded the mutation data available to patients with TCGA-HNSC from the TCGA Data Portal (https://portal.gdc.cancer.gov/). Somatic mutation data is stored in mutation annotation format (MAF), and we analyze mutation data from HNSCC samples using maftools (Mayakonda et al., 2018). We calculated the individual tumor mutation burden (TMB) score for each HNSCC patient and explored the relationship between risk score and TMB. The TMB score is calculated as follows: (Total Mutation/Total Coverage Base) × 10^6 (Robinson et al., 2017).

### 2.7 Drug sensitivity

The treatment response of patients in the high- and low-risk groups was assessed using the pRRophetic R software package, which was determined by each HNSCC patient in Cancer Drug Susceptibility Genomics (GDSC) (https://www.cancerrxgene.org/)and Cancer Therapeutics Response Portal (CTRP) (https://portals.broadinstitute.org/ctrp/) determined by the semi-maximum inhibitory concentration (IC50) (Geeleher et al., 2014).

### 2.8 Statistical analysis

Statistical analysis is carried out using R software v4.1.3. *p*-values < 0.05 are considered statistically significant, and FDR (false detection rate) q < 0.05 is considered statistically significant.

## 3 Results

### 3.1 Identification of candidate NFRGs

**FIGURE 1:**
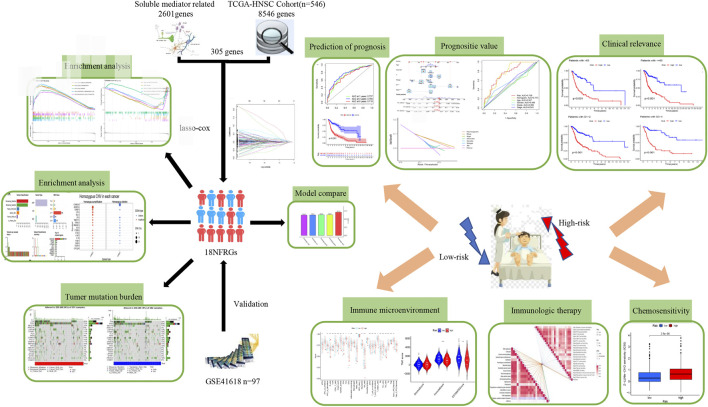
Workflow of the study.

**FIGURE 2:**
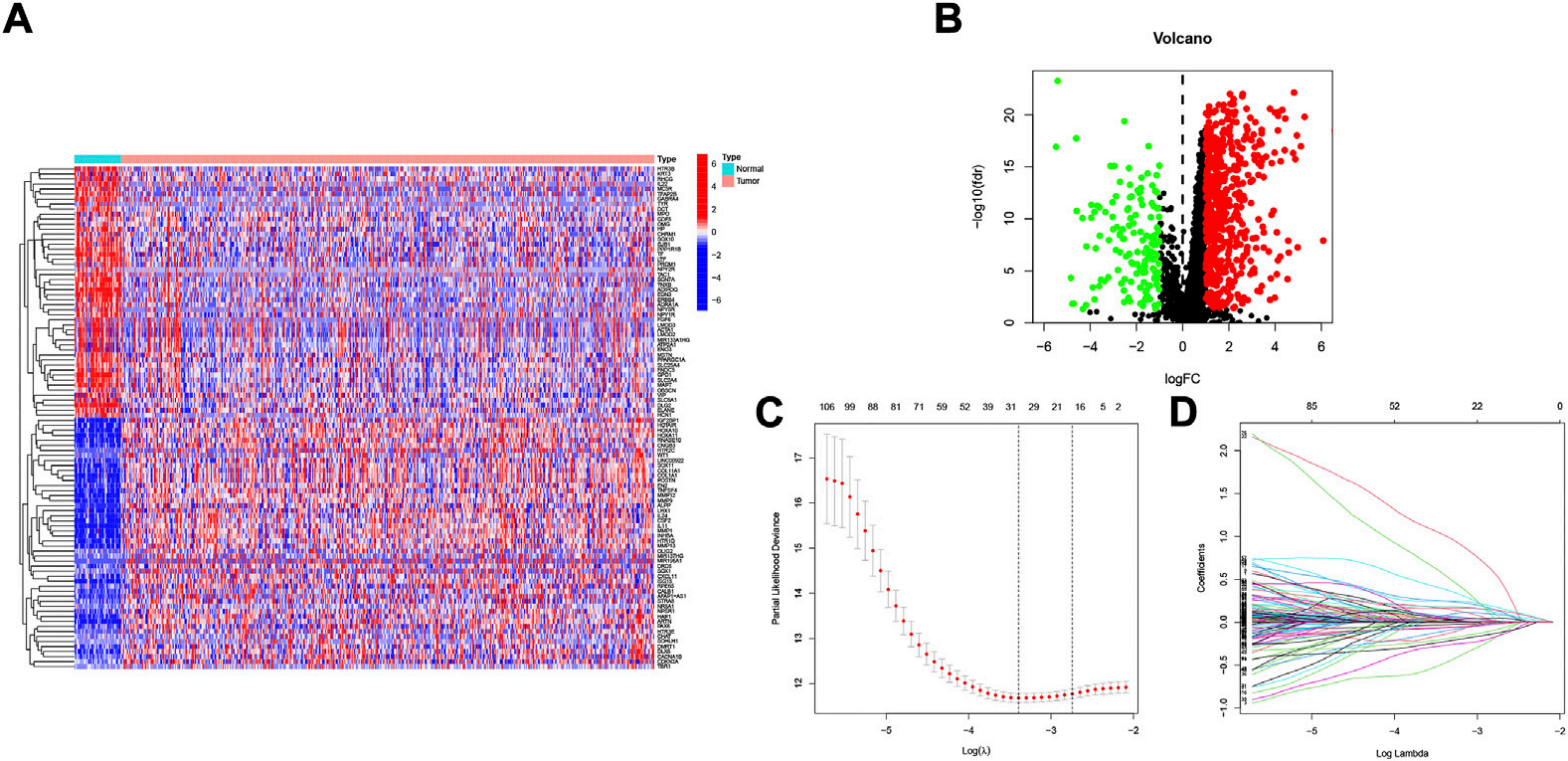
Identification of candidate NFRGs. **(A)** Heat map of the difference in mRNA expression between tumor samples and normal samples. **(B)** Volcano map of NFRGs with differential expression. **(C)** Adjustment of parameters and **(D)** cross-validation in the LASSO model.

Figure 1 shows the flow chart of the study protocol. To find biomarkers that can effectively predict the prognosis of HNSCC, we developed a risk score model based on neurotrophic factor-related genes to assess the prognosis of HNSCC patients. Clinical information and mRNA expression of 546 HNSCC samples were collected and downloaded from The Cancer Genome Atlas (TCGA). The gene set of neurotrophic factors was obtained from the Genecard database, which contains 2601 genes. A heat map was created based on the difference in mRNA expression between tumor samples (*n* = 502) and normal samples (*n* = 44) (Figure 2A). The differential expression analysis of genes based on |log2FC|>0.5 was performed on HNSCC tumor tissues by applying the “limma” R package, and 562 genes with up-regulated expression and 152 genes with down-regulated expression were obtained (Figure 2B). We performed univariate Cox analysis of differentially expressed NFRGs by the “survival” R package and extracted 305 prognostically relevant NFRGs (*p* < 0.05). Next, we subjected these 305 NFRGs to lasso regression analysis and obtained 31 NFRGs (Figures 2C,D), and further downscaled these high-dimensional data by a multifactorial Cox proportional risk regression model, and finally identified 18 NFRGs, namely TGFB1, IL10, CDKN2A, ADIPOQ, EPO CHAT, LPL, TAC1, CTSG, CYP2D6, DES, RNASE3, PGK1, SFRP1, TRIB3, TMEFF2, GRIA3, and EFNB2. And the corresponding regression coefficients coef were obtained as 0.2934, −0.9684, −0.0727, 0.3209, −0.3963, 0.3167, 0.1622, 1.5022, −0.1324, −0.4854, 0.0532, 0.8230, 0.3546, −0.0934, 0.3191 0.9016, −0.4756 and 0.2471. In multivariate Cox analysis, the linear prediction model was built based on 18 NFRGs weighted by their regression coefficients. 18 NFRGs weighted by their correlation coefficients were given by the formula: risk score as = (0.2934 × TGFB1 expression level) + (−0.9684 × IL10 expression level) + (−0.0727 × CDKN2A expression level) + (0.3209 × ADIPOQ expression level)+(−0.3963 × EPO expression level) + (0.3167 × CHAT expression level) + (0.1622 × LPL expression level) + (1.5022 × TAC1 expression level) + (−0.1324 × CTSG expression level)+(-0.4854 × CYP2D6 expression level) + (0.0532 × DES expression level) + (0.8230 × RNASE3 expression level) + (0.3546 × PGK1 expression level) + (−0.0934×SFRP1 expression level) + (0.3191 × TRIB3 expression level) + (0.9016 × TMEFF2 expression level) + (−0.4756 × GRIA3 expression level) + (0.2471 × EFNB2 expression level).

### 3.2 Validating the accuracy of the NFRGs model to predict patient prognosis

**FIGURE 3:**
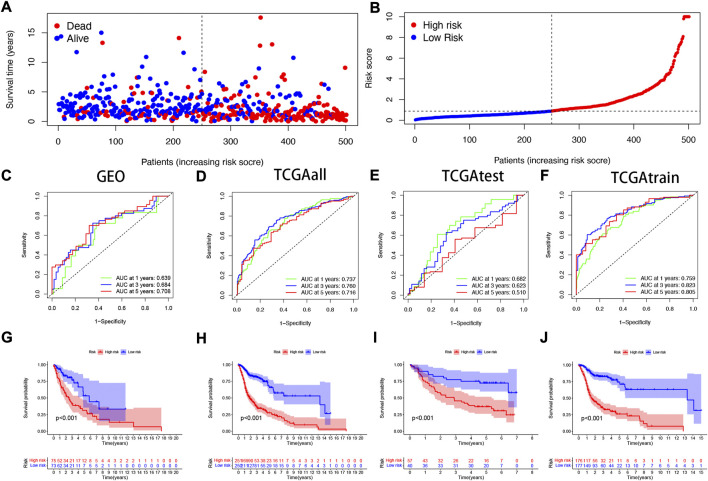
Validating the accuracy of the NFRGs model to predict patient prognosis **(A,B)** Partial likelihood deviation map. Time-dependent ROC curve of HNSCC patients **(C)** in the GEO cohort; **(D)** in the TCGA all cohort; **(E)** in the TCGA test cohort; **(F)** in the TCGA train cohort. K-M survival curve of HNSCC patients **(G)** in the GEO cohort; **(H)** in the TCGA all cohort; **(I)** in the TCGA test cohort; **(J)** in the TCGA train cohort.

To verify the accuracy of the prognostic model we constructed, patients included in the study (n = 546) were randomly divided into training cohorts and test cohorts (train:test = 7:3). In the training cohort, mortality in surviving HNSCC patients increased with increased risk (Figures 3A,B). We then constructed a time-dependent receiver operation characteristics (ROC) curve and found that both the ROC curve of the GEO cohorts and the ROC curve of the TCGA cohorts show that the performance of the prognostic signature we constructed is very prominent (Figures 3C–F). At the same time, the survival curve was constructed to analyze the prognosis differences between the high-risk and low-risk groups, and it was found that the prognosis of high-risk patients was worse than that of low-risk patients in both test and training cohorts (*p* < 0.001).

### 3.3 PCA correlation analysis

**FIGURE 4:**
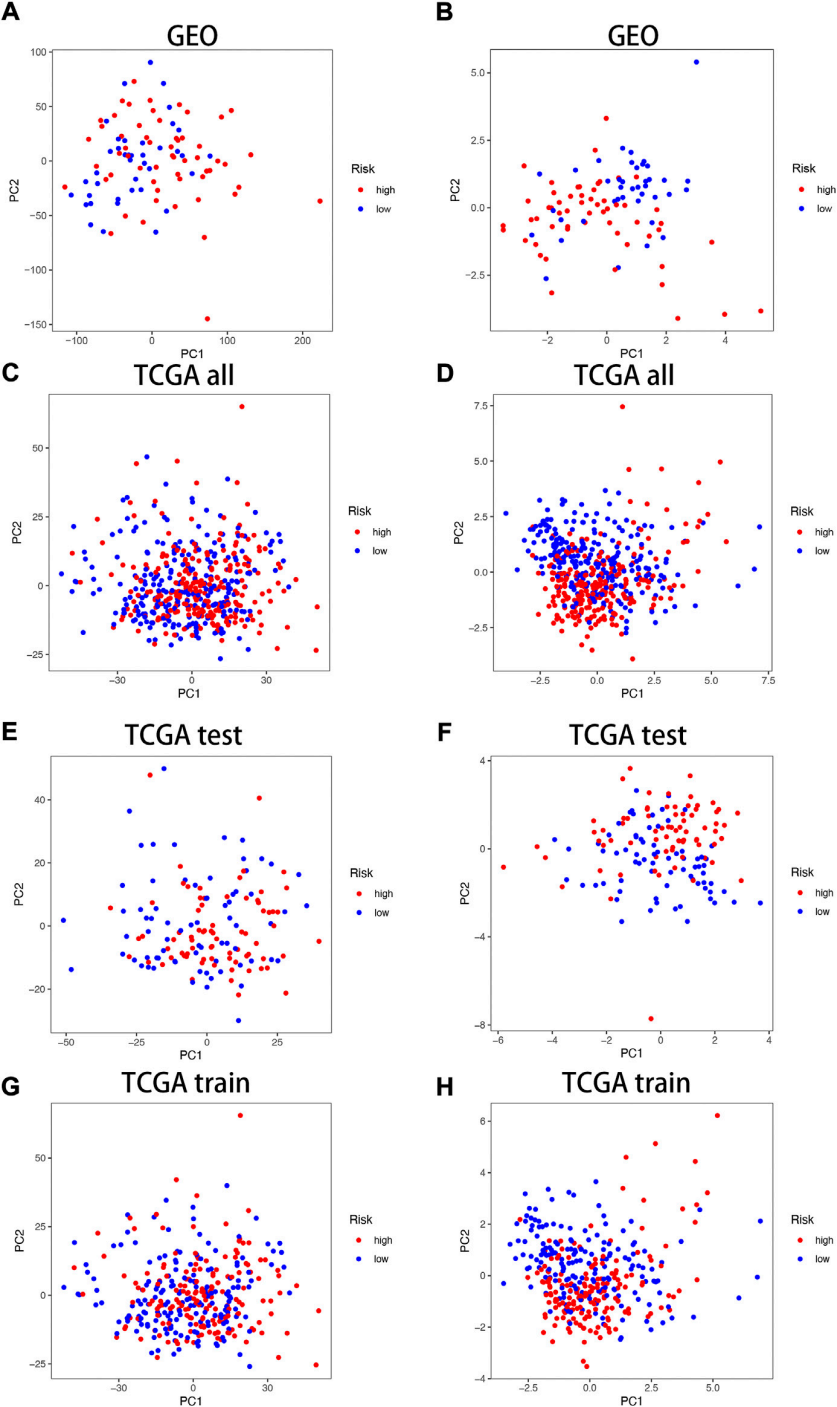
PCA correlation analysis in TCGA and GEO Cohorts. In GEO Cohort: **(A)** PCA plots of neurotrophic factor genes; **(B)** PCA plots of model genes. In TCGA Cohort: **(C)** PCA plots of neurotrophic factor genes in the sum group; **(D)** PCA plots of model genes in the sum group; **(E)** PCA plots of neurotrophic factor genes in the test group; **(F)** PCA plots of model genes in the test group; **(G)** PCA plots of neurotrophic factor genes in the training group; **(H)** PCA plots of model genes in the training group.

In the TCGA and GEO cohorts, we divided the samples into high and low-expression groups based on median risk scores, respectively, and then performed PCA analysis based on model genes *versus* neurotrophic factor-related genes to obtain PCA plots of neurotrophic factor genes *versus* model genes in the GEO cohorts (Figure 4A,B) and the TCGA cohorts for the sum group (Figures 4C,D), test group (Figures 4E,F) and training group (Figures Figure4G,H) of the neurotrophic factor genes with the PCA plot of the model genes (Figures 4C,D). The results showed that the high-risk and low-risk groups were most clearly differentiated among the model gene groups.

### 3.4 Combining clinical characteristics to build nomograms

**FIGURE 5:**
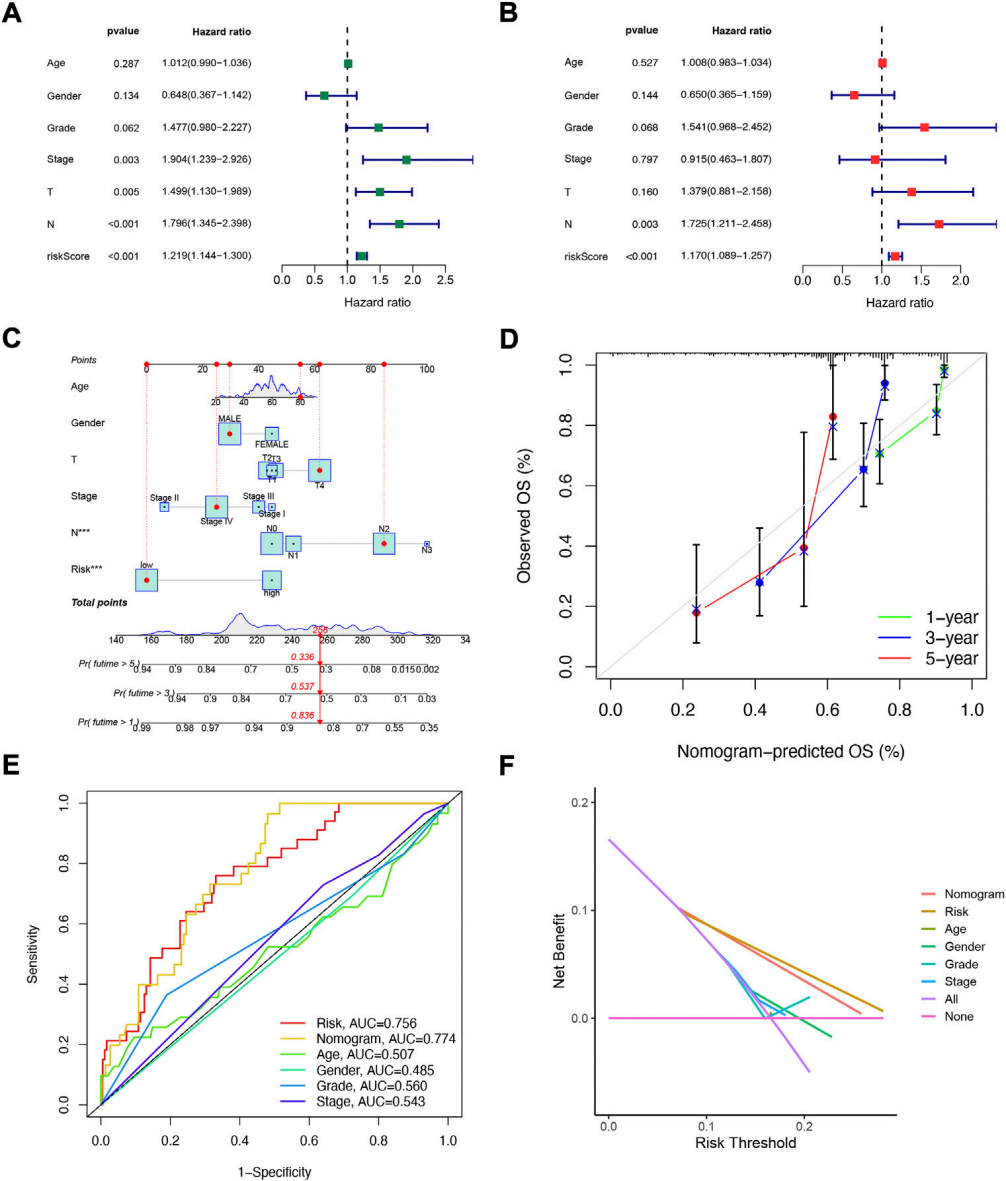
Independent prognostic analysis of risk scores and clinical parameters. Univariate **(A)** and multivariate **(B)** COX regression analysis of the signature and different clinical features. **(C)** Nomogram for predicting 1-year, 3-year, and 5-year OS of patients with HNSCC. **(D)**The calibration curve of the constructed nomogram of 1- year, 3- year, and 5-year survival. **(E)** Multi-index ROC analysis in the test cohort. **(F)** Decision curve analysis.

Considering that the constructed risk model of NFRGs was significantly associated with the prognosis of HNSCC patients, to further determine whether the prognostic characteristics constructed based on the 18 NFRGs could be used as an independent factor to predict prognosis, we combined the OS of HNSCC patients with their clinical characteristics for univariate and multivariate Cox analyses. According to the results of univariate analysis, T (*p* = 0.005), N (*p* < 0.005), Stage (*p* = 0.003), and risk score (*p* = 0.003) were significantly associated with the prognosis of HNSCC patients (Figure 5A). Subsequent multifactorial Cox analysis was performed, and the risk score remained a reliable, independent risk predictor (*p* < 0.001) (Figure 5B). To expand the clinical application and usability of the constructed NFRGs risk model for HNSCC, we constructed nomograms based on age, grade, stage, T, N, and risk score as a means of predicting 1-, 3-, and 5-year prognostic survival probabilities. In addition, the model results showed that the risk score had the greatest influence on predicting OS and also indicated that the risk model based on 18 NFRGs genes could better predict the prognosis of head and neck squamous cell carcinoma (Figure 5C). The calibration curves also showed satisfactory agreement between predicted and observed values in terms of the probability of 1-year, 3-year, and 5-year OS (Figure 5D). The NFRGs risk score model (AUC = 0.756) was more predictive of HNSCC prognosis than the traditional age and tumor grading and clinicopathological characteristics (Figure 5E). Consistent with this result, our model had the highest net benefit, indicating that our NFRGs risk model is more influential in clinical decision-making (Figure 5F).

### 3.5 Correlation analysis of NFRGs risk scores with clinicopathological features

**FIGURE 6:**
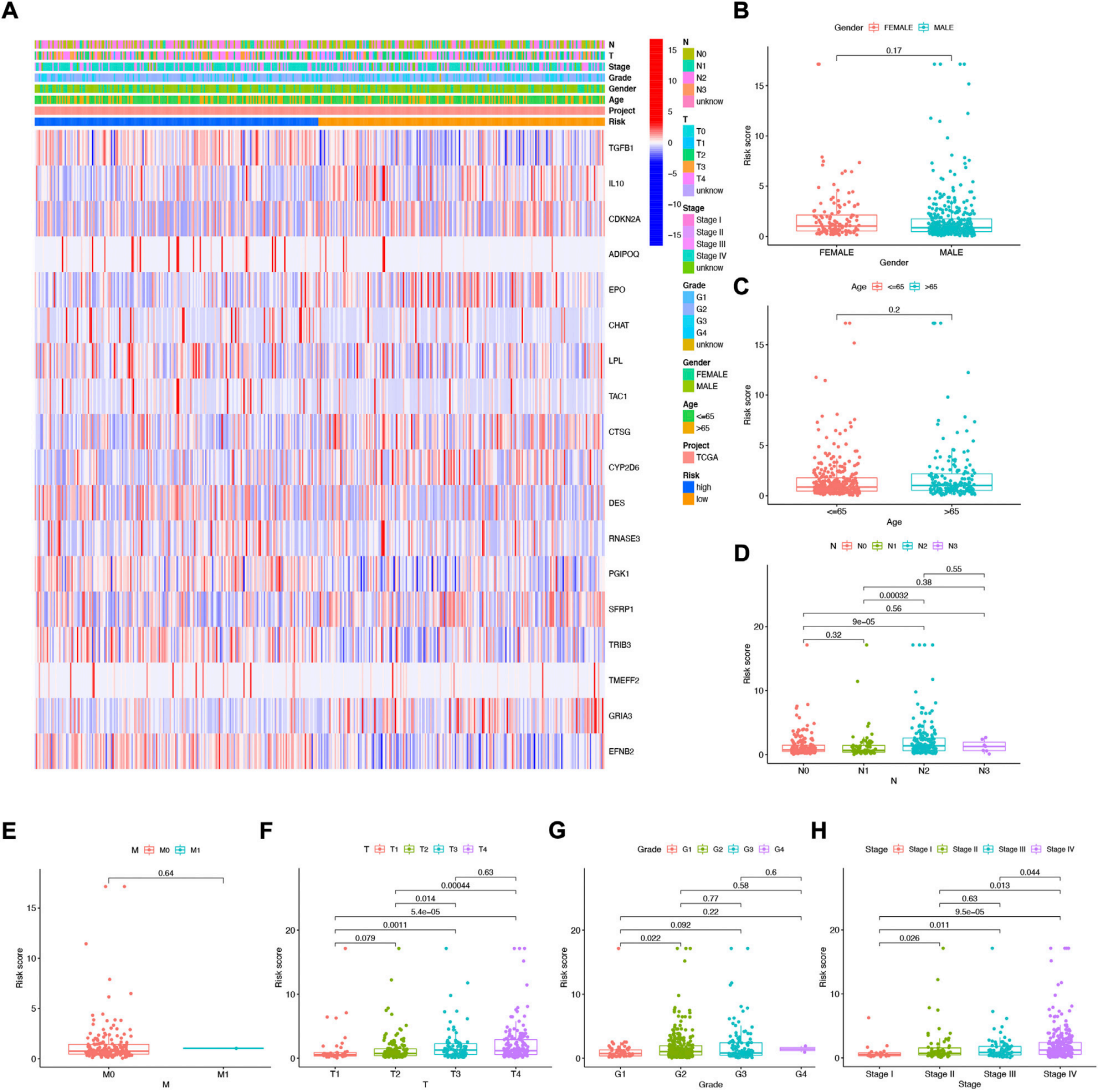
Correlation analysis of risk scores and clinicopathological features and signatures based on 18 NFRGs **(A)** heat maps **(B)** gender, **(C)** age, **(D)** N stages, **(E)** M stages, **(F)** T stages, **(G)** tumor grades, **(H)** pathological stages.

The heat map shows the association between the gender, age, grade, stage, T, N, and risk score of 18 NFRG genes found in the prognostic risk model and the samples of all head and neck squamous cell carcinoma patients in the TCGAs (Figure 6A). At the same time, to examine the correlation between the risk model and the clinical pathological characteristics of patients with HNSCC, the risk score of each subgroup was compared by the Wilcoxon test in terms of age, tumor grade, stage, T stage, M stage, N stage, and gender. The results showed that the risk score was significantly correlated with tumor grade (*p* < 0.05), T stage (*p* < 0.05), and stage (*p* < 0.05) but not with age, M stage, N stage, and gender. (Figures 6B–H).

### 3.6 Clinical subgroup analysis of the NFRGs risk model

**FIGURE 7:**
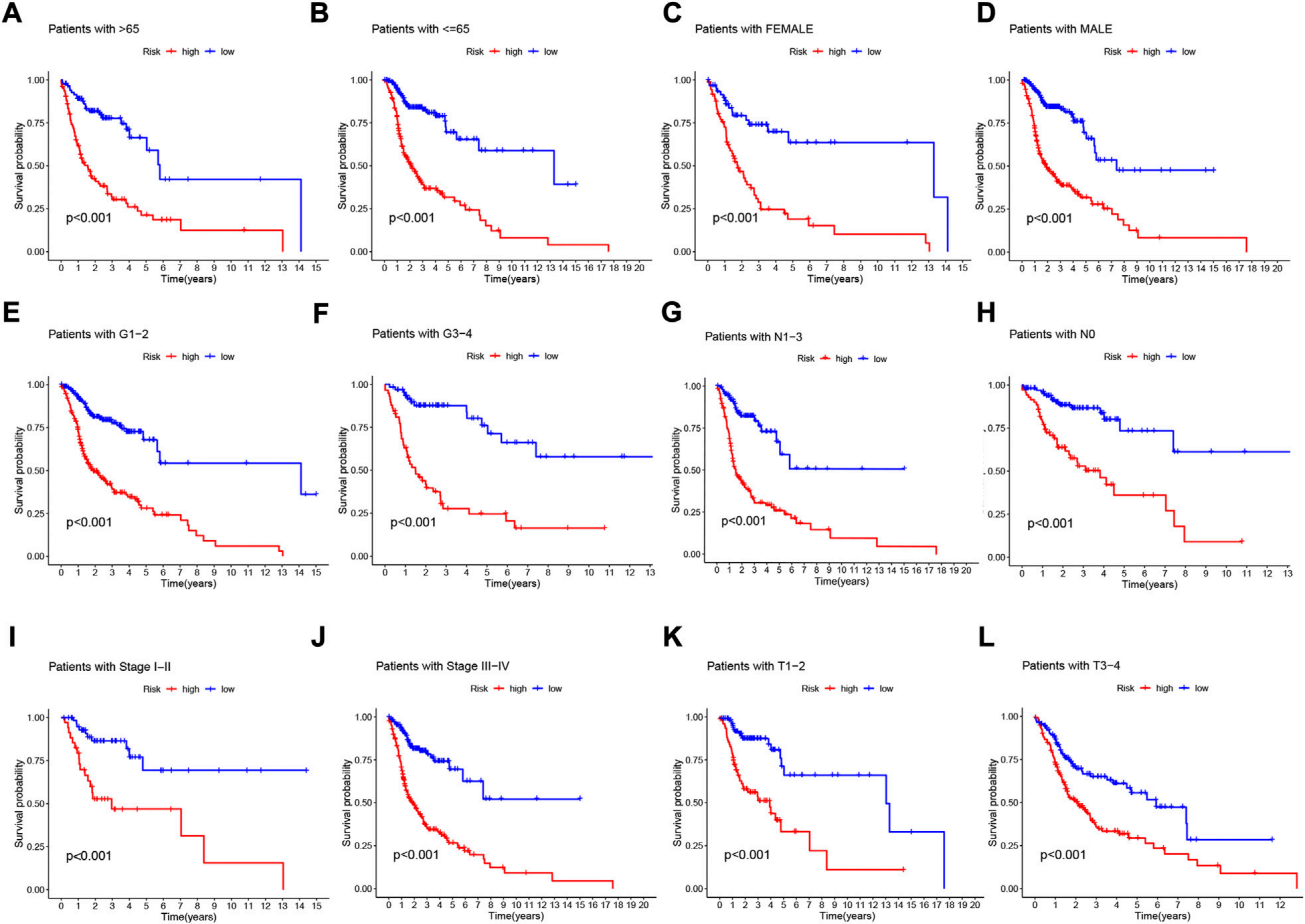
Prognostic power of the NFRGs risk model for overall survival for multiple HNSCC subtypes. **(A)** Age >65 years. **(B)** Age≤65 years. **(C)** Female. **(D)** Male. **(E)** Grade I-II. **(F)** Grade III-IV. **(G)** N0. **(H)** N1-3. **(I)** Stage I-III. **(J)** Stage III-IV. **(K)** T1-2. **(L)** T3-4.

To further understand whether there are differences in the prognosis of patients in different clinical subgroups, we collated clinical data from the entire TCGA sample. Subsequently, the samples were divided into different subgroups according to age (>65 and≤65 years), gender (male and female), tumor grade (grade I-II and III-IV), pathological N stage (N0 and N1-3, pathological stage (I-III and III-IV) and pathological T stage (T1-2 and T3-4) for further stratified survival analysis (Figure 7). The results showed that in all subgroups, patients in the high-risk group had significantly lower OS than the low-risk group **(** Figures 7A–L). These results suggest that our NFRGs risk model also has a reliable predictive value for the prognosis of different clinical subgroups of HNSCC.

### 3.7 NFRGs signature performs better than other signatures in prognosis prediction

**FIGURE 8:**
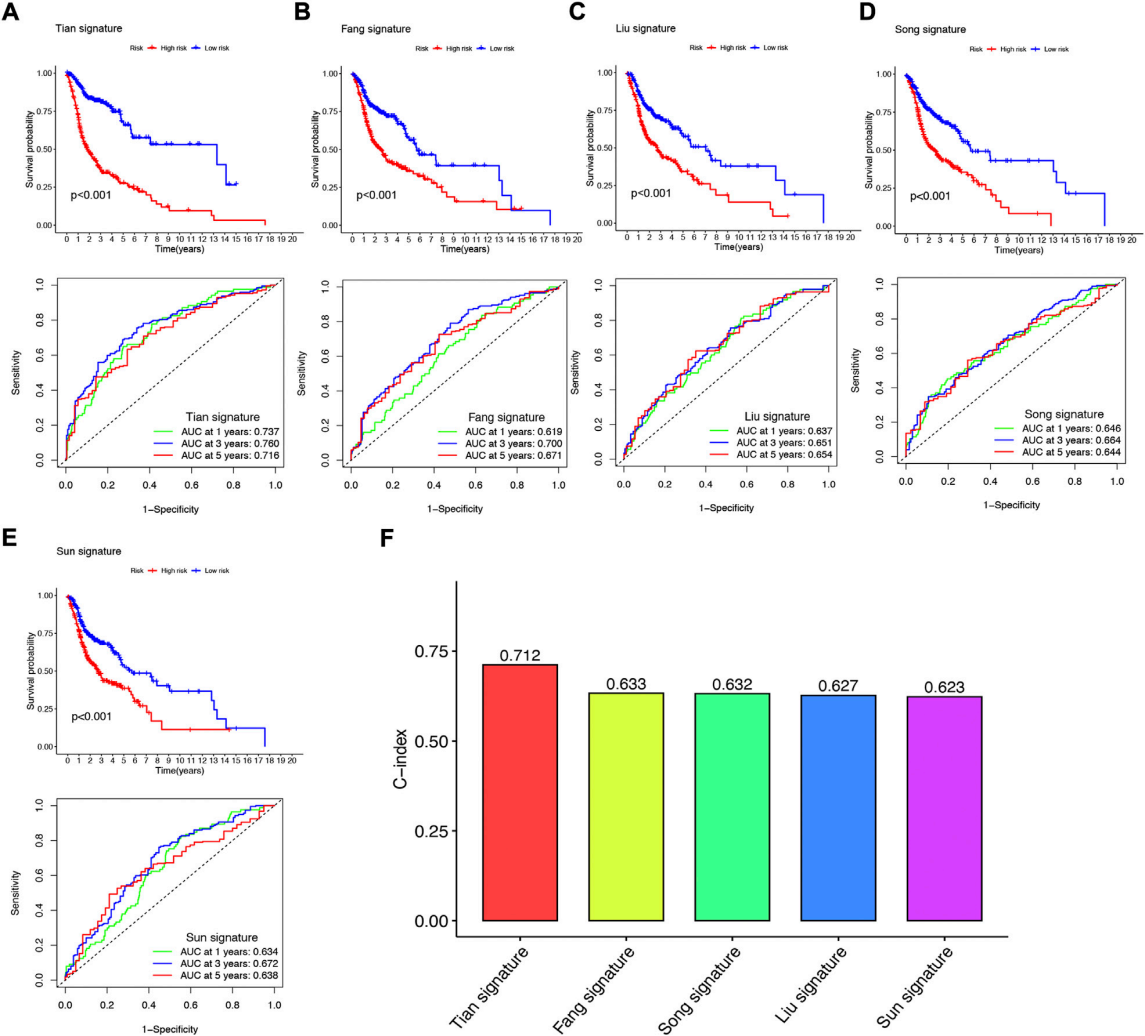
Comparison of the NFRGs signature with other models **(A)** KM curves and ROCs for NFRGs signature. **(B–E)** KM curves and ROCs for risk models constructed by others. **(F)** C-indexes for five risk models.

To further demonstrate whether our constructed NFRGs signature has accurate predictive power for HNSCC patients, we compared it with four published prognostic signatures, namely the Fang signature, Liu signature, Song signature, and Sun signature. To ensure the comparability of the signatures, we calculated risk scores for each HNSCC sample in the entire TCGA cohort using the same method and transformed the risk scores across the four signatures according to the previous method. Although these four signatures effectively divided HSNCC patients into two subgroups with significantly different prognoses, time-dependent ROC curve analysis showed that these four signatures had lower AUC values at 1-, 3-, and 5-year survival than our NFRGs signature (Figures 8A–E). In addition, Figure 8F shows that our NFRGs signature had the highest C-index (AUC = 0.712). All these results suggest that our constructed NFRGs signature has a more prominent predictive performance.

### 3.8 NFRGs risk score predicts tumor microenvironment (TME) and immune cell infiltration

**FIGURE 9:**
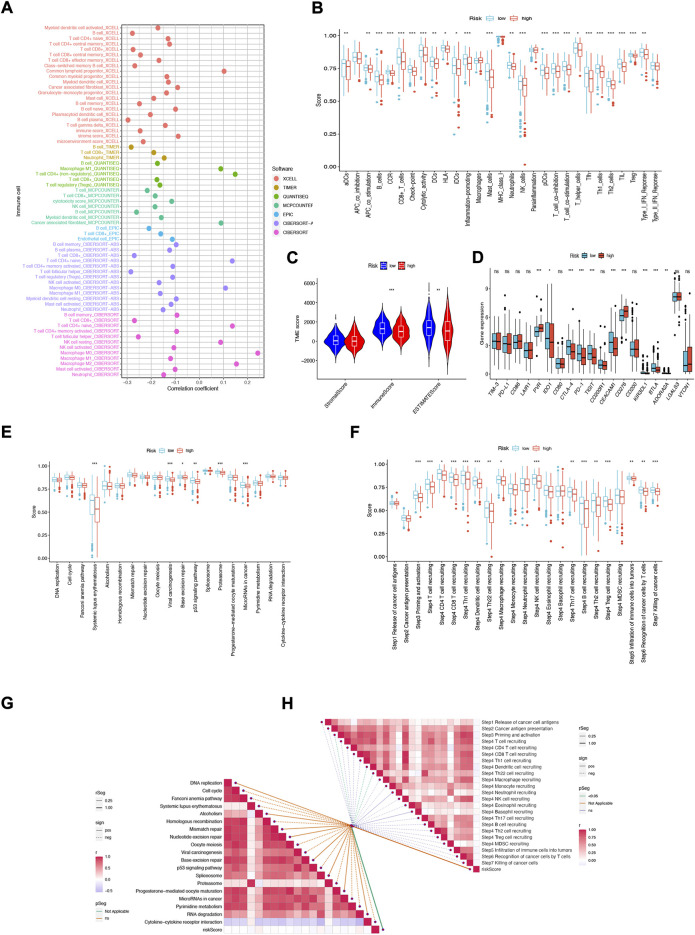
NFRGs risk score predicts tumor microenvironment and immune cell infiltration. **(A)** Immune cell bubble plots. **(B)** Immune cell and immune function ssGSEA scores between high and low-risk groups. **(C)** TME component analysis. **(D)** Immune checkpoint differences between high- and low-risk groups. **(E)** ICB response signature differences between high and low-risk groups. **(F)** Differences in immune steps with tumor between high- and low-risk groups. **(G)** Correlation between risk score and ICB response signature. **(H)** Correlation of risk scores with each step of the tumor immunization cycle. *p < 0.05; **p < 0.01; ***p < 0.001.

Immune features of TME include the expression levels of immune checkpoint inhibitors (ICIs), infiltration of tumor-infiltrating immune cells (TIICs), and activity of the cancer immune cycle (Fan et al., 2021). First, we investigated the risk score based on XCELL, TIMER, QUANTISEQ, MCPCOUNTER, CIBERSORT, CIBERSORT- ABS, and EPIC algorithms and explored the correlation between risk score and infiltrating immune cell abundance (Figure 9A). Subsequently, we performed a comparison of one-sample GSEA (ssGSEA) scores for immune cells and immune function, with the vast majority of immune cells and immune function scoring significantly greater in the low-risk group than in the high-risk group (Figure 9B).

The results suggest that this NFRGs risk score model may significantly inhibit or enhance the expression of specific immune cell types and immune function, thus affecting the response to immunotherapy. In addition, as infiltrating immune cells are an important component and one of the characteristics of the tumor microenvironment (TME), changes in the expression of immune cell types can lead to changes in TME composition, so we analyzed the TME composition of HNSCC samples using ESTIMATE. The results showed that the immune score (*p* < 0.001) as well as the ESTIMATE score (*p* < 0.01) were higher in the low-risk group compared to the high-risk group, indicating that the overall immune level and immunogenicity of the tumor microenvironment were higher in the low-risk group (Figure 9C). Given the importance of checkpoint-based immunotherapy, further differences in immune checkpoint expression were found between the two groups. Eight immune checkpoint genes were found to be significantly upregulated in the low-risk group, including IDO1, CTLA-4, PD-1, TIGIT, CEACAM1, KIR3DL, and BTLA. ADORA2A (Figure 9D). Based on these results, it can be suggested that risk scores can guide clinicians in the use of immune checkpoint-targeted drugs. Since the immune microenvironment mediates ICB responses, we further analyzed the differences in ICB response signatures between high and low-risk groups and found that in the low-risk group, Systemic lupus erythematosus, Viral carcinogenesis, Base excision repair, p53 signaling pathway, Proteasome, and microRNAs in cancer risk scores were higher in the low-risk group than in the high-risk group, and there were no significant differences in other ICB response signatures **(** Figure 9E). Meanwhile, the correlation between NFRGs risk scores and ICB-related positive signatures was analyzed, and no significant correlation was found between them (Figure 9G). Subsequently, to further refine the immune profile of the HNSCC tumor microenvironment, we also performed a differential analysis of tumor immune step risk scores between high and low-risk groups. In the low-risk group, upregulation of activity was observed for most steps in the cycle, including priming and activation (step 3), transport of immune cells to the tumor (step 4) (T-cell recruiting, CD4 T-cell recruiting, CD8 T-cell recruiting, Th1 cell recruiting, DC cell recruiting, Th22 cell recruiting, macrophage recruiting, NK cell recruiting, Th17 cell recruiting, B-cell recruiting, Th2 cell recruiting, Treg cell recruiting), Infiltration of immune cells into tumors (Step 5), Recognition of cancer cells by T cells (Step 6), Killing of cancer cells (Step 7) (Figure 9F). Simultaneous correlation analysis between risk score and tumor immune cycle steps revealed that only priming and activation (step 3), DC cell recruiting, and Th22 cell recruiting were significantly negatively correlated with risk score (Figure 9H).

### 3.9 Mutation analysis and biological functional enrichment analysis

**FIGURE 10:**
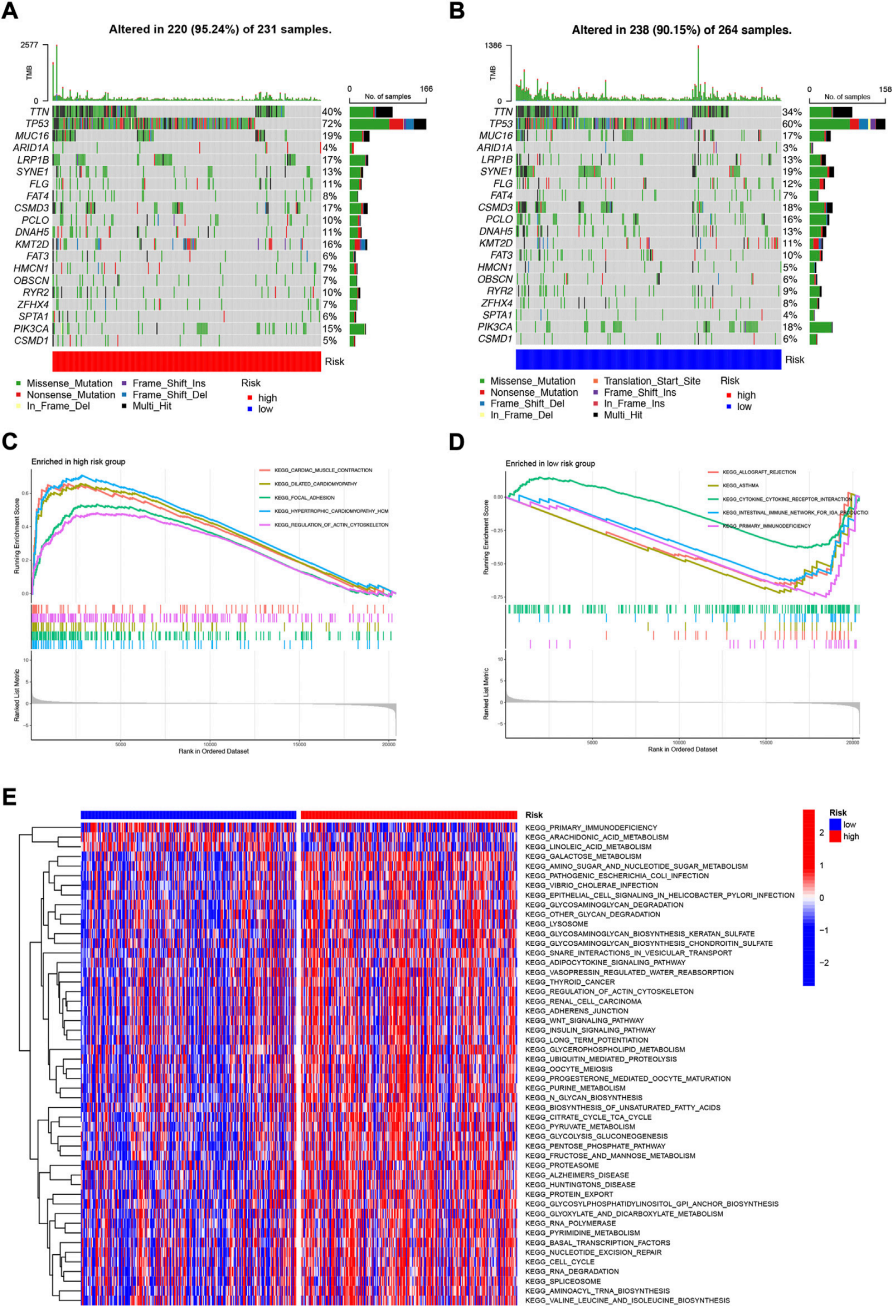
Mutation analysis and biological function enrichment analysis **(A)** Mutation analysis of high-risk group **(B)** Mutation analysis of low-risk group **(C)** Enrichment pathway of high-risk significantly up-regulated gene set **(D)** Enrichment pathway of low-risk significantly down-regulated gene set **(E)** Heat map of difference in enrichment scores between high- and low-risk groups.

We analyzed and visualized somatic mutation data from HNSCC patients by distinguishing between high-risk and low-risk groups. The top three mutated genes in high-risk patients were TP53 (72%), TTN (40%), and MUC16 (19%); the top three mutated genes in low-risk patients were TP53 (60%), TTN (34%), and SYNE1 (19%) (Figures 10A,B). It has been shown that different mutational statuses and expression patterns of wild type may lead to different clinical outcomes of the immune response, with wild-type TP53 patients having a higher sensitivity to radiotherapy for HNSCC (Cao et al., 2019). In addition, TP53 mutations are more likely to occur in HPV-negative HNSCC and less common in HPV-positive HNSCC (Helman et al., 2014), possibly suggesting that TP53 acts as an indicator of radiotherapy sensitization target and HPV typing in patients with HNSCC, which has great value for clinical studies. To elucidate the potential biological pathways associated with our risk genes, we performed Gene set enrichment analysis (GSEA) (Figures 10C,D) and Gene Set Variation Analysis (GSVA) using the Kyoto Gene and Genome Encyclopedia (KEGG) pathway database on risk group samples (Figure 10E) for Kyoto genes; the results showed that highly activated gene sets in the high-risk group were associated with RNA polymerization and degradation as well as cell cycle, cancer-related pathways. We subsequently obtained pathways that were significantly enriched in the high and low-risk groups. Among them, the expression of gene sets associated with primary immunodeficiency pathways was significantly downregulated in the low-risk group. These functional enrichment results also confirm the correlation between the immune microenvironment and gene expression differences analyzed in the previous sections.

### 3.10 Multi-omics mutation characteristics and drug susceptibility analysis of NFRGs

**FIGURE 11:**
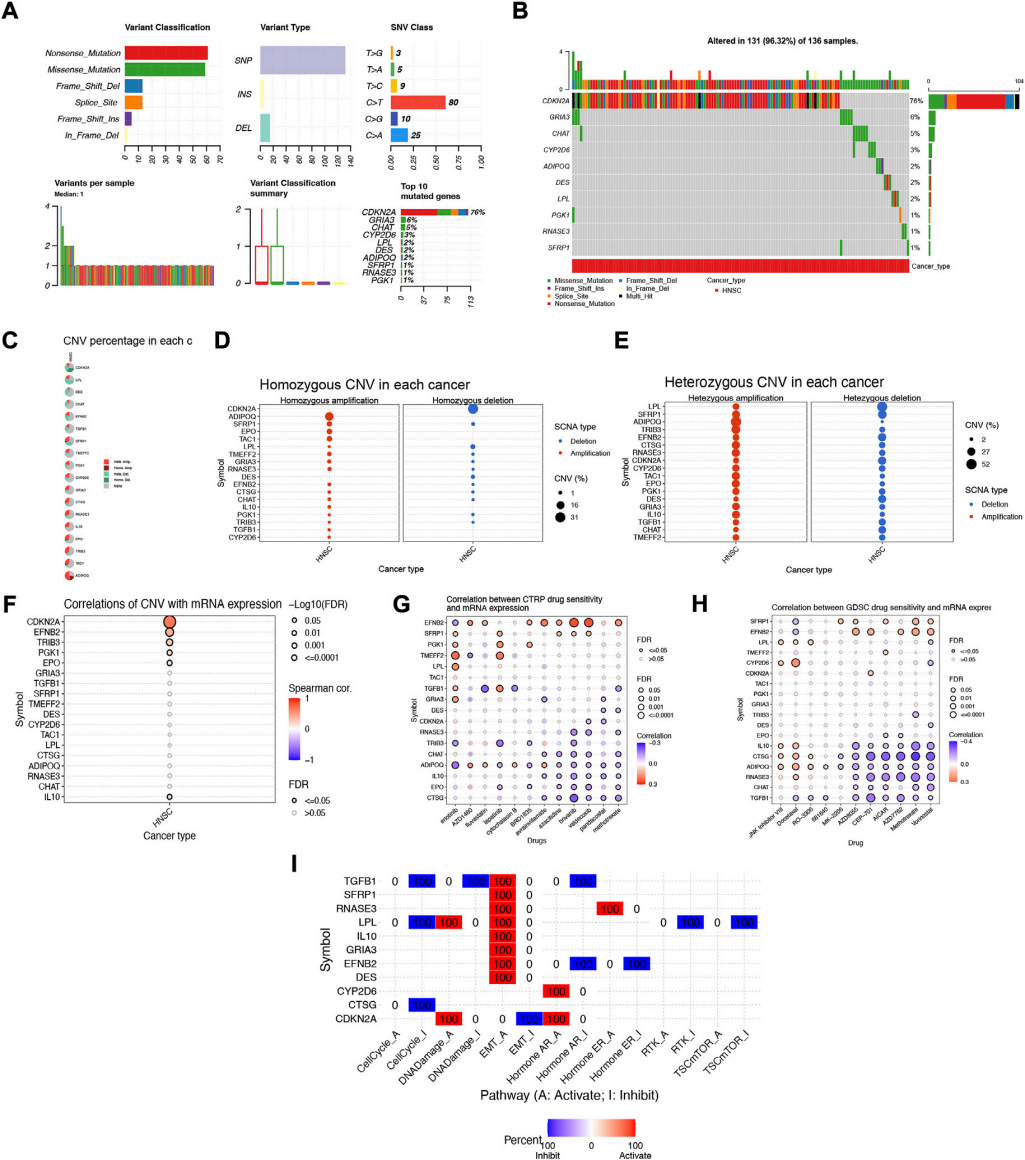
Multi-omics mutation characteristics and drug sensitivity analysis of NFRGs. **(A,B)** Classification of mutations in HNSCC and mutation incidence of NFRGs. **(C)** The proportion of different types of copy number variation in NFRGs. **(D and E)** The distribution of copy number variant amplification and deletion in homozygous mutations versus heterozygous mutations. **(F)** Correlation analysis of copy number variation and expression of NFRGs. **(G,H)** Correlation analysis of expression of NFRGs with the sensitivity of chemotherapeutic drugs in CTRP and GDSC cohorts. **(I)** Analysis of the role of expression activity of NFRGs in the regulation of cancer-related pathways.

To further explore the biological mechanism of abnormal expression of these 18 target genes, we analyzed them from different omics levels such as genome level and copy number level. Single nucleotide site variation (SNV) results showed that the Nonsense_Mutation of NFRGs was the most common variant classification in the TCGA-HNSC cohort, while the most prevalent variant type was single nucleotide polymorphism (SNP). Compared to other SNV categories, C>T has the highest frequency **(** Figure 11A). And the mutation occurred in 131 patients with HNSCC, with CDKN2A having the highest mutation frequency (Figure 11B). Subsequently, the analysis of copy number variation (CNV) was carried out to summarize the ratio of homozygous mutations to heterozygous mutations in NFRGs copy number variations in the sample (Figure 11C), In addition, we counted the two mutations separately, and the results showed that the amplification of homozygous mutations in the sample was mainly ADIPOQ, while CDKN2A was mainly characterized by copy number deletion, and the amplification of heterozygous mutations was mainly ADIPOQ, while the LPL was mainly copy number deletion (Figures 11D,E). In addition, the Speedman correlation coefficient analysis between copy number variation and gene expression was carried out, and it was found that the copy number variation of IL10 was down-regulated in HNSCC, while CDKN2A, EFNB2, TRIB3, PGK1, EPO were upregulated (Figure 11F), Therefore, abnormal gene expression may be the result of a combination of single nucleotide variation and copy number variation. In addition, we obtained significant correlations between the expression differences of NFRGs and the drug sensitivity of the Cancer Therapeutics Response Portal (CTRP) and Genomics of Drug Sensitivity in Cancer (GDSC) databases **(** Figures 11G,H). This means that the expression of our risk profile genes can be used as a predictor of drug sensitivity to chemotherapy in patients or as a target for future drug sensitization. Finally, we explore the relationship between the expression of NFRGs and the activity of cancer-related pathways. It can be seen that under the regulation of 18 genes, the cell cycle, RTK, and TSCmTOR pathways of patients with HNSCC are inhibited, while the DNA-Damage, EMT, apoptosis, Hormone AR, and Hormone ER pathways are activated or inhibited (Figure 11I).

### 3.11 TIDE and drug susceptibility analysis based on NFRGs

**FIGURE 12:**
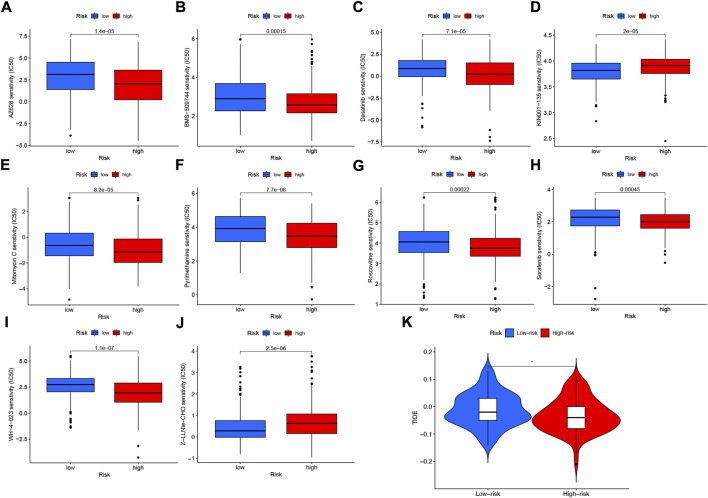
Differences in IC50 of immunotherapy drugs by risk score **(A)** AZ628, **(B)** BMS-509744, **(C)** Dasatinib, **(D)** KIN001-135, **(E)** Mitomycin C, **(F)** Pyrimethamine, **(G)** Roscovitine, **(H)** Sorafenib **(I)** WH-4-023 **(J)** Z-LLNle-CHO . **(K)** TIDE score differences between high- and low-risk groups. *p < 0.05; **p < 0.01; ***p < 0.001.

Among the 10 immunotherapeutic agents applied in the treatment of HNSCC, the low-risk group included AZ628 (*p* = 1.4e-05), BMS-509744 (0.00015), Dasatinib (*p* = 7.1e-05), Mitomycin C (*p* = 8.2e-05), Pyrimethamine (*p* = 7.7e- 06), Roscovitine (*p* = 0.00022), Sorafenib (*p* = 0.00045), WH-4-023 (*p* = 1.1e-07), IC50 were higher compared to the high-risk group (Figures 12A–C,E–I). In addition, we found that two other chemical or targeted drugs, KIN001-135 (P = 2e-05), and Z-LLNIe-CHO (*p* = 2.5e-06), had lower IC50 in the low-risk group (Figures 12D,J). Based on the risk score, we can further study the immunotherapy response of patients with HNSCC and enhance precise drug therapy. In addition, we use the Tumor Immunocompromise and Exclusion (TIDE) algorithm to predict the likelihood of immunotherapy risk models. The TIDE in the low-risk group was significantly higher than those in the high-risk group (*p* < 0.05) (Figure 12K), indicating that the higher the likelihood of immune evasion in the low-risk group, suggesting that patients were less likely to benefit from ICI (immune checkpoint inhibitor) therapy.

## 4 Discussion

HNSCC is a common malignancy caused by abnormal squamous cells. With more research on HNSCC, the role of nerves in the development of tumorigenesis has been reflected, in which neurotrophic factors are involved in the mutual communication between cancer cells and the nervous system to promote tumor progression and gradually be concerned (Cervantes-Villagrana et al., 2020). Perineural invasion (PNI) and perineural spread (PNS) are considered to be the critical links of tumor growth and metastasis (Albo et al., 2011; Roh et al., 2015; Rademakers et al., 2017). Some studies have shown that cancer cells stimulate the growth of nerve fibers by secreting neurotrophic factors, thus completing PNI and PNS. What is exciting is that the growing nerve fibers can also promote tumor growth and cancer cell proliferation, thus forming positive feedback (Lu et al., 2017; Zhang et al., 2022c). Neurotrophic factors are also widely studied in HNSCC. Many clinical studies have shown that the local recurrence rate of patients with PNI is 23–36%, while that of patients without PNI is 9–5% (Fagan et al., 1998; Tai et al., 2013; Pinto et al., 2014). Another study showed that TrkB, as a high-affinity receptor for BDNF and NT-4, is highly expressed in HNSCC and that TrkB receptor blockers can inhibit the proliferation of cancer cells *in vitro* (Kupferman et al., 2010; Dudás et al., 2011). At the same time, the interaction between BDNF and TrkB is also believed to regulate tumor cell invasion and drug resistance, leading to poor prognosis. It may be the action mechanism of TrkB receptor blockers (Dudás et al., 2019). However, there is a lack of systematic study of the value of the neurotrophic factor family in predicting tumor prognosis.

This study constructed a polygenic model based on neurotrophic factor-related genes. Subsequently, we conducted a validation analysis of the constructed NFRGs risk scoring model and found that it can effectively assess the prognosis of patients with HNSCC. The risk score of each patient was calculated based on the expression levels of the 18 NFRGs screened out, and the risk group was divided into high and low-risk groups according to the median risk score. The nomogram was then constructed in combination with clinical pathological factors, and the calibration curve showed a satisfactory agreement between the predicted and observed values in terms of 1-year, 3-year, and 5-year OS. At the same time, with traditional clinical indicators such as age, sex, tumor grade, histological staging, *etc.*, the prognosis of HNSCC can be predicted. Taken together, our model has the highest net return, suggesting that our NFRGs risk model is more influential in clinical decision-making, and clinicians can tailor anti-tumor personalized treatment based on nomogram results.

In our modeling genes, it has been shown that transforming growth factor β-inducible protein (TGFB1) can inhibit tumor progression by promoting apoptosis (Skonier et al., 1992; Zhao et al., 2002). It has also been proposed that TGFB1 may influence the behavior of oral squamous cell carcinoma through mechanisms such as involvement in tumor fibrosis, epithelial-mesenchymal transition (EMT), and extracellular matrix remodeling (Donohoe et al., 2017; Hu et al., 2019), but few studies have reported on the role of TGFB1 in HNSCC. We note that HNSCC can promote Th2-skewed response by regulating IL-10 expression and secretion in the tumor microenvironment (Jewett et al., 2006; Young, 2006; Johnson et al., 2014) and that IL-10 has been shown to inhibit IFN-α production in HNSCC (Caruntu et al., 2022), which may lead to antitumor poor therapeutic efficacy. In addition, based on mouse models, CDKN2A could inhibit p53R172H-induced metastasis in HNSCC, and patients with HNSCC with both high-risk p53 mutations and pure CDKN2A deletions had the worst clinical outcomes (Li et al., 2016). Erythropoietin (EPO) is commonly thought to alleviate anemia in patients after radiotherapy. However, clinical trials have demonstrated worse tumor control in HNSCC patients treated with EPO and found that EPO can promote lymphatic tract metastasis in HNSCC through mediated activation of JAK-STAT signaling, thereby enhancing tumor aggressiveness, which is detrimental to patient prognosis (Lai et al., 2005). The mechanism of action of other NRFGs in HNSCC remains to be elucidated.

Extensive characterization of TME is crucial for establishing reliable prognostic markers and new advanced modern HNSCC treatment regimens (Elmusrati et al., 2021); we are very interested in immune function and expression of immune cells in the tumor microenvironment, so we conducted immune cell infiltration, TME components, ssGSCA, and other analysis, and found that the low-risk group was higher than the high-risk group in terms of immune cells and immune function. This suggests that our risk model can distinguish the cold-heat tumor subtype from patients with HNSCC and suggests that the hot tumor subtype has a better prognosis.

Immune checkpoints have attracted much attention as one of the important features of TME. Some clinical studies have shown that immune checkpoint inhibitors (ICI), such as Nivolumab and Pembrolizumab, have good antitumor effects in HNSCC (Qiang et al., 2021). By using monoclonal antibodies against immune checkpoints (ipilimumab against CTLA-4, or nivolumab and pembrolizumab against PD1), cancer immunotherapy effectively releases tumor-induced immune system brakes to restart cancer immune circulation (Tan et al., 2017). However, the heterogeneous phenotypes present in HNSCC exhibit different genetic aberrations in complex mutational environments, which makes their response to targeted therapies limited (Elmusrati et al., 2021). According to previous clinical trials, the response rate of recurrent or metastatic HNSCC to PD-1/PD-L1 inhibitors was only 13.3–22%. Therefore, it is crucial to select patients who can respond effectively to ICIs (Guo et al., 2021). The analysis of differences in immune checkpoint activity between high and low-risk groups showed that NFRGs models were able to distinguish patients with differences in important immune checkpoint activity, and using these immune checkpoints as targets for immunotherapy may lead to better immunotherapy outcomes, providing guidance for decision-making before clinical immunotherapy. Among them, programmed death ligand 1 (PD-L1) as an immune checkpoint protein in the cancer immune cycle is highly expressed in the low-risk group, which may indicate that tumor cells in low-risk patients rely on the PD-1/PD-L1 signaling pathway to evade immune monitoring, and PD-1 monoclonal antibodies may have a good effect on patients in the low-risk group. Upregulation of inhibitory immune checkpoints such as PD-1 is a key feature of inflamed TME (Spranger et al., 2013), which may imply that low-risk patients are in an inflammatory microenvironment. In addition, we found that CD276 was highly expressed in the high-risk group, upregulated in HNSCC and helped tumor cells evade immune surveillance (Li et al., 2022), consistent with our predicted results. In 4-nitroquinoline-induced mouse HNSCC, cancer stem cells (CSCs) use the immune checkpoint molecule CD276 (B7-H3) to evade immune surveillance (Elmusrati et al., 2021). Since mRNA expression profile data from HNSCC patients receiving immunotherapy was not available, the potential of this signature to predict immunotherapy responses was indirectly assessed, which could lead to deviations from the actual situation. Therefore, in the future, it should be validated in conjunction with data from HNSCC patients receiving immunotherapy.

Our NFRGs risk scoring model is a good predictor of prognosis for patients with HNSCC, and nomograms based on this model can help clinicians personalize treatment for HNSCC. Experimental studies of neurotrophic factor-related molecular mechanisms and related clinical cohort studies can be carried out in the future, which have great clinical value and may provide a reliable direction for precision medicine.

## Data Availability

The datasets presented in this study can be found in online repositories. The names of the repository/repositories and accession number(s) can be found in the article/supplementary material.
